# Spatial Distribution of Cutaneous Leishmaniasis Cases Referred to Health Centers of Three Khorasan Provinces in Iran Using Geographical Information System

**Published:** 2019-10

**Authors:** Mohammad Reza SHIRZADI, Mohammad JAVANBAKHT, Nahid JESRI, Abedin SAGHAFIPOUR

**Affiliations:** 1. Communicable Diseases Management Center, Ministry of Health and Medical Education, Tehran, Iran; 2. Department of Remote Sensing and GIS, School of Geography, University of Tehran, Tehran, Iran; 3. Remote Sensing & GIS Centre, Shahid Beheshti University, Tehran, Iran; 4. Department of Public Health, Faculty of Health, Qom University of Medical Sciences, Qom, Iran

**Keywords:** Cutaneous leishmaniasis, Spatial analysis, Geographic information system, Iran

## Abstract

**Background:**

Nowadays, geographic information system (GIS) is one of the most useful epidemiological tools for identifying high-risk areas of cutaneous leishmaniasis. The aim of this study was to determine the spatial distribution of cutaneous leishmaniasis in northeastern Iran.

**Methods:**

In this cross-sectional study, information on positive cases of cutaneous leishmaniasis in the three provinces located in northeastern Iran during Jul 2011 to Jul 2017 was obtained from the Iranian Ministry of Health. Based on the postal address of each case, the geographical coordinates of each patient were determined for spatial analysis of cutaneous leishmaniasis. For spatial analysis, Moran's index autocorrelation and Kriging interpolation method were used in GIS software.

**Results:**

Moran's index autocorrelation showed that spatial distribution of disease incidence in the study area was cluster pattern (Z-score > 1). In addition, Kriging interpolation method revealed that 90% of southern parts of North Khorasan province and northern parts of Razavi Khorasan Province formed hot spots.

**Conclusion:**

The CL incidence is a function of spatial and geographical trends. In addition, spatial trends in the disease incidence distribution indicate that it is not greatly increased or decreased from one area to another. It appears as hot spots areas. Spatial analysis by showing high risk areas can be useful tools for controlling and preventing CL incidence.

## Introduction

Cutaneous leishmaniasis (CL) is spreading in most areas of the world (1). The disease is prevalent in 102 tropical and subtropical countries (2). The geographical distribution of the disease is dependent on the distribution of its vectors; the sand flies (3). The annual new cases of CL in the world is 1.5 to 2 million, with more than 90% of the disease occurring in Afghanistan, Algeria, Iran, Iraq, Saudi Arabia, Syria, Brazil and Peru (4). This disease is observed in more than half of Iran's 31 provinces as endemic foci and there are most of studies, which they are shown epidemiological aspects of CL such as reservoirs hosts and human infections of the disease in all parts of Iran (5–7). Iran's geographic and climatic conditions are suitable for the growth and proliferation of rodents and sandflies as reservoirs and vectors of this disease (8).

The CL incidence is increasing in the world, because of environmental changes that caused by humans, such as uncontrolled exploitation of wood resources and deforestation, mining, sedimentation, agricultural extension, new irrigation methods, development of roads within forests as well as massive migration from rural to urban areas and urbanization. Furthermore, poverty and malnutrition are additional factors that contribute to disease incidence. Generally, the risk factors for the disease are expressed based on parameters such as age, sex, economic conditions, and other social factors (9). The distribution of CL in Iran is not similar and there are some foci in different regions of Iran. Identifying risk foci of the disease can be effective in managing and controlling the disease and adopting its preventive programs (10).

Currently, GIS is one of the most important and epidemiological tools in determining the high-risk areas of zoonotic diseases, including CL (11). This software can be useful in identifying the geographical areas and populations at risk of the disease. Because of this, in planning for the provision of prevention and monitoring the disease in terms of temporal and special can be effective. Geographic information system is applicative software that can provide a spatial distribution pattern of diseases, including CL, to study the relevant or probable effective factors on prevalence of the disease. It can show map out the spatial distribution of each disease quantitatively and qualitatively in form of mapping (12). One of the practical applications of GIS is helping to make decisions about health management and preventing strategies and control of various diseases, including CL, malaria and HIV infection (13). The main objective of this study was to determine the spatial distribution of CL incidence in northeastern Iran using GIS software during 2011–2017.

## Materials and Methods

### Study area

**Fig. 1:**
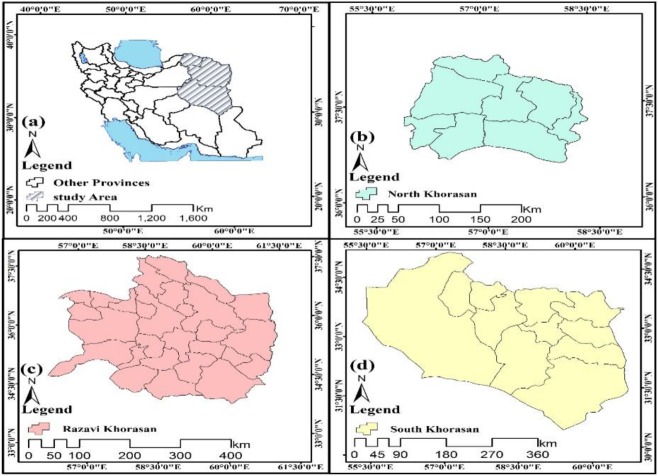
Location map of the study area.

One of the main foci of CL in Iran is northeastern areas of the country (5,14). Therefore, the study area in this study was selected from three northeastern provinces of Iran: North Khorasan, Razavi Khorasan, and South Khorasan (Fig. 1).

Based on the general census of population and housing in 2016, the population of three provinces was 863092, 6434501, and 768898 people in North Khorasan, Razavi Khorasan and South Khorasan respectively (15). Their total areas are also 28311, 118854, and 148669 km^2^, respectively (Fig. 1).

### Data collection

In order to determine the temporal and special changes in CL incidence, the data of people with disease in three provinces in northeastern Iran; of North Khorasan, Razavi Khorasan and South Khorasan over a period of six years, from 2011 to 2017 of Centers for Disease Control and Prevention (CDC) of the Iran Ministry of Health and Medical Education was received. All suspected patients with skin lesion(s) referred to regional health centers of the province were examined for CL using clinical and parasitological tests. Samples were taken from the borders of the suspected lesion, fixed with methanol, stained with Geimsa, and examined under the microscope. The disease was diagnosed based on clinical examination and microscopic observation of the intracellular amastigotes of the parasite in the Geimsa stained smear.

### Data analysis

#### Cutaneous leishmaniasis incidence

Firstly, in order to determine the spatial changes of CL, the incidence of disease was estimated based on population. For spatial analysis, the distribution of CL was carried out using two analyzes: Global Index of Spatial Autocorrelation - Moran's I and Hot Spot Analysis in GIS software.

*Global Index of Spatial Autocorrelation - Moran's I* This index is one of the most practical and most important analytical tests on spatial data. The Z-score for this index is variable in the range of 1 to −1. If the Z-score of Moran autocorrelation index is greater than zero, the spatial distribution of disease has cluster pattern, the Z-score is smaller than zero, the spatial distribution of disease has random pattern and Z-score equal to zero, the of spatial distribution of the disease will have to scatter plot.

### Hot Spot Analysis

Hot spot analysis is an appropriate method for investigating the distribution of geographic phenomena in space. This analysis shows in which areas large or small amounts of data are distributed clustery. In other words, this method analyzes each complication in the context of the complications in its neighborhood. In order to make a complication as hot spot and statistically be significant, in addition to it, the complications that are in its neighborhood must have also high amounts. In this analysis, the local amounts of a complication and its neighbors are compared with the sum of the total complications, and if the local amounts increase unexpectedly and this increase is such that this increase cannot be considered because of randomization, these phenomena form hot spots.

In the analysis of hot spots using Getis- Ord Gi in ArcGIS software, the randomized distribution of CL in three provinces: North Khorasan, Razavi Khorasan and South Khorasan in northeastern Iran was assumed at a significant level of 0.05 and bandwidth 200 km tested. In this present study, Microsoft Office Excel 2017 software was used for data entry, and to perform the tests. ArcGIS v.9.3 was used to mapping and analyzes hot spots. Furthermore, some climatic and environmental variables) in northeastern provinces of Iran were gathered. Table 1 shows the mean of six climatic and environmental variables (rainfall, temperature, vegetation, relative humidity, evapotranspiration, and soil moisture) in northeastern provinces of Iran.

**Table 1:** Mean of climatic and environmental variables and cutaneous leishmaniasis incidence in northeastern provinces of Iran

***Index***	***North Khorasan***	***Razavi Khorasan***	***South Khorasan***
Rain precipitation rate (mm)	282.56	259.22	183.22
Near surface air temperature (°c)	13.49	16.02	20.56
Normalized Difference Vegetation Index (NDVI)	0.19	0.13	0.08
Relative humidity (%)	42.94	34.60	19.11
Evapotranspiration (kg/ m2 -y)	255.44	233.99	156.10
Soil moisture (%)	11.71	11.99	10.01

## Results

### Special and temporal distribution of cutaneous leishmaniasis

The average CL incidence rate was estimated in three provinces over six years, 53.07/100000 people. In addition, the average incidence rate in North Khorasan, Khorasan Razavi, and South Khorasan during the six years was 81.25, 69.49 and 8.46/100,000 people respectively. Investigation of spatial trends in CL incidence based on Moran autocorrelation index and hot spots analysis showed that the northern areas had a higher incidence than southern areas. Therefore, the disease incidence had the northern-southern trend increased from south to north. In addition, statistical analysis showed that the incidence of disease was higher in southern regions of North Khorasan Province and north of Razavi Khorasan Province. The incidence rate was relatively lower in southern regions of Razavi Khorasan Province and in the throughout South Khorasan Province.

### Global Index of Spatial Autocorrelation - Moran's I

**Fig. 2:**
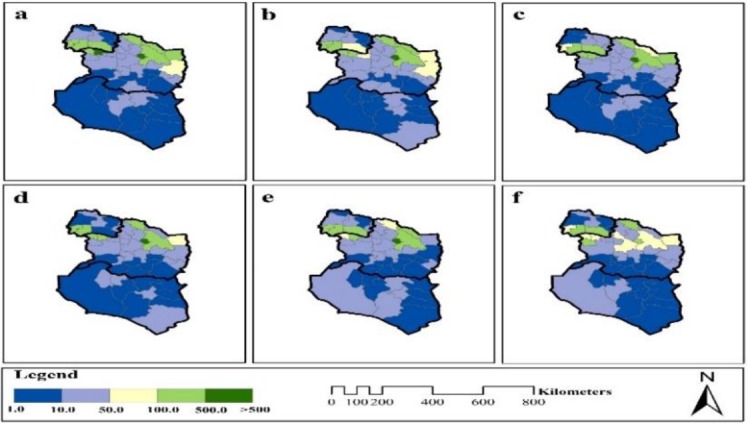
Spatial Autocorrelation-Moran's I plotted on the study area map and overlaid with cutaneous leishmaniasis incidence in northeastern Iran during 2011–2017

**Fig. 3:**
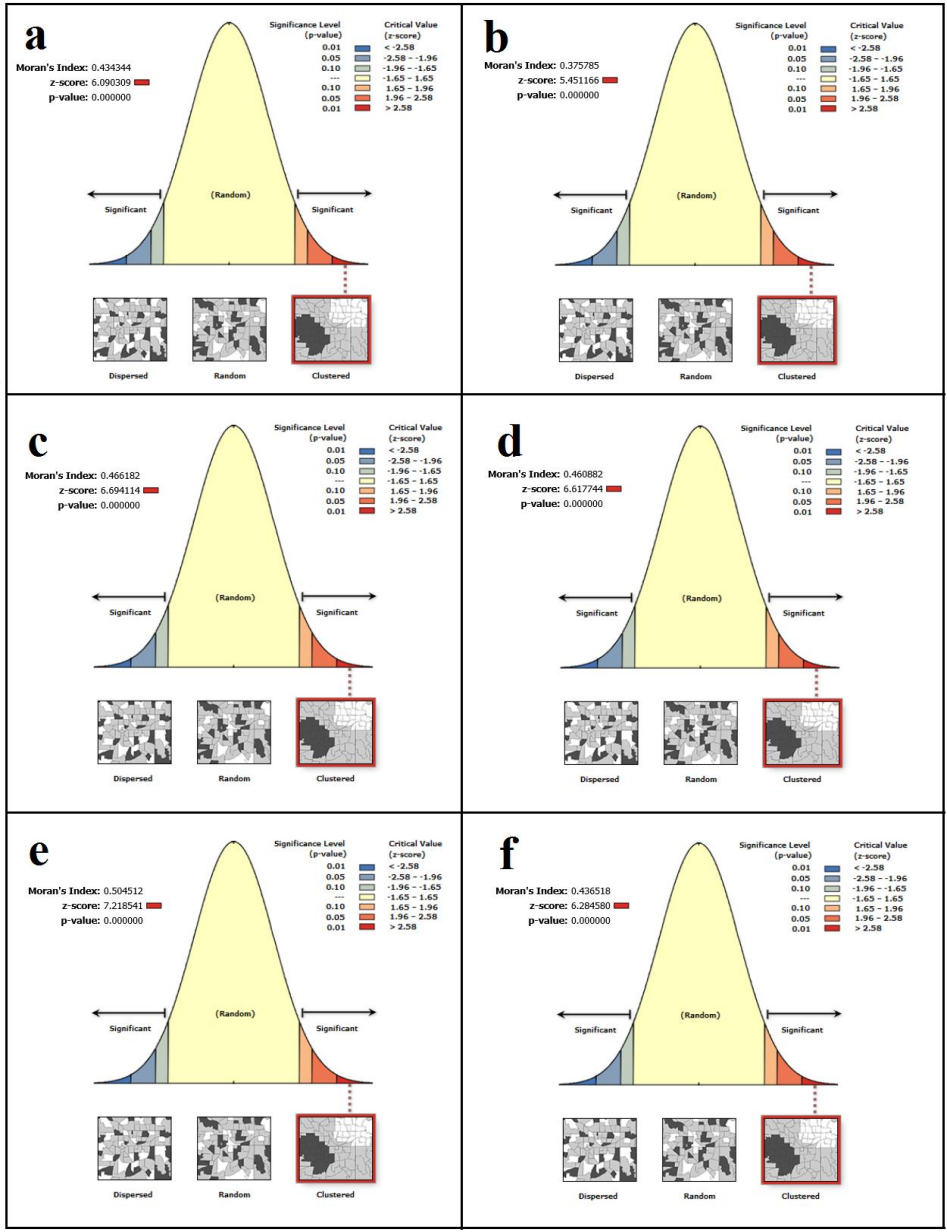
Spatial Autocorrelation-Moran's I for cutaneous leishmaniasis incidence in northeastern Iran during 2011–2017

Regarding the high incidence of disease in the southern regions of North Khorasan and northern areas of Razavi Khorasan, spatial autocorrelation - Moran's I index was used to determine the spatial pattern of the disease. Autocorrelation coefficients for different years are given in Fig. 2. As the coefficients are deductible, Moran index shows Z-score was greater than zero for all years. This means that spatial distribution of disease incidence in the study area has cluster pattern (Z-score > 1) (Fig. 3). On the other hand, the areas where adjacent to each other, have similar or close proximity to the disease and form spatial clusters. Because of *P*-value (chance of randomness of observations) is zero and Z-Score is greater than 2.5, the cluster pattern of the disease incidence in the study area is not random and the adjacent regions are spatially correlated. Therefore, the assumption of the randomness of the spatial distribution of CL incidence was rejected.

### Hot Spot Analysis

**Fig. 4:**
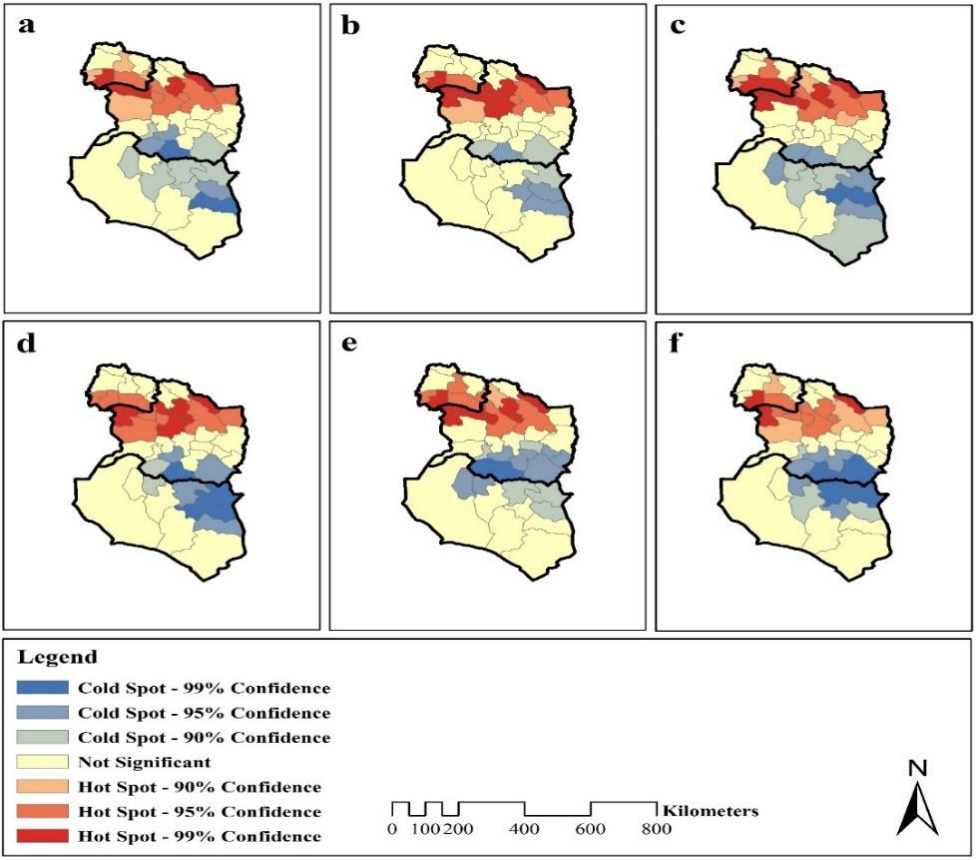
Hot & Cold-Spot maps of Cutaneous Leishmaniasis in Northeast of Iran from 2011 to Jul 2017

Moran correlation results showed that there is a cluster pattern in the incidence of CL in the studied area. $G_{i}^{*}$ index was used to identify the range and position of high or low spatial clusters of the disease. The results of this analysis on spatial distribution of the disease incidence showed that 90% of southern parts of North Khorasan Province and northern parts of Razavi Khorasan Province formed hot spots. In addition, the southern and southeastern parts of Razavi Khorasan Province and the east of South Khorasan Province formed cold spots (Fig. 4).

## Discussion

According to spatial modeling and Spatio-temporal analysis of cutaneous leishmaniasis in Iran, distribution of susceptible regions in terms of CL incidence in Iran, the main foci of the disease in Iran were Isfahan, North Khorasan, Razavi Khorasan, Markazi, Fars, South Khorasan, Kerman, Qom, Tehran, Qazvin, Semnan provinces respectively (5,14,16,17). According to present study, the average incidence rate in North Khorasan, Khorasan Razavi and South Khorasan during the six years was 81.25, 69.49 and 8.46/100,000 people respectively. Moreover, this study on Moran autocorrelation index and hot spots analysis revealed that spatial trends in CL incidence-based showed that the northern areas had a higher incidence than southern areas. Therefore, the disease incidence had the northern-southern trend and increased from south to north.

In addition, the incidence of disease was higher in southern regions of North Khorasan Province and north of Razavi Khorasan Province. The incidence rate was relatively lower in southern regions of Razavi Khorasan Province and in the throughout South Khorasan Province. It means spatial distribution of disease incidence in the study area has cluster pattern. The results of this analysis on spatial distribution of the disease incidence showed that 90% of southern parts of North Khorasan Province and northern parts of Razavi Khorasan Province formed hot spots.

The activity, growth of sand flies as vectors of CL are more affected by climatic factors such as soil moisture, evaporation and transpiration, vegetation indices, humidity, precipitation, and to some extent of air temperature (18). The incidence of CL disease depends on sand flies' density. Therefore, CL is indirectly affected by climatic factors. The incidence of the disease depends on a variety of factors in addition to the population of sandflies. For example, the change in population and the density of the disease reservoirs (rodents) can affect the incidence of disease. At the time of drought, the population of zoonotic rodents approaching human settlements may increase. This, in turn, could increase the population of CL infected rodents as well as the incidence of the disease because of these rodents being bitten by the sand flies and subsequent bating on humans and inoculation of the *Leishmania* parasite to humans. Ultimately, it leads to an increase the disease incidence.

We found that the average air temperature and relative humidity in the North Khorasan, Razavi Khorasan and South Khorasan provinces was 13.49 °C & 42.94%, 16.02 °C & 34.60% and 20.56 °C & 19.11% respectively. The growth of all species of sand flies occurs at temperatures above 18 °C, which is the threshold temperature for their life. In general, the optimum temperature for the development of their life stages before maturation of sand flies is 28–28 °C (19). In the Rajasthan region of India, the thermal threshold for the growth of sand flies was 35–17 °C (20). Adult sand flies activate during sunset and night when the air temperature is balanced and humidity rises. In this condition, the infected female phlebotomine sandflies can cause the transmission of *Leishmania* parasites into susceptible hosts such as humans.

The relative humidity is favorable for egg hatching, being about 80%. Sink et al. also found 30% relative humidity as the threshold for the survival of the sandflies as vectors of CL. Furthermore, relative humidity of 31%–85% was desirable for sand flies (20). Hence, in the present study area, climatic conditions are favorable for the activity of sand flies in terms of humidity, their biting habit on hosts such as humans and the transmission of CL disease. Although relative humidity is required in breeding places of sand flies, high moisture can make larvae migrate to other places (19).

Therefore, the temperature and humidity conditions for sand flies and the disease incidence in southern parts of North Khorasan Province and northern parts of Razavi Khorasan Province were more appropriate. This study was designed on registered data in health center of Qom province, Iran. This makes the results of this study, can state just some epidemiological aspects of CL patients in three Khorasan Provinces in Iran. In our knowledge this is can be one of the limitations of this the study.

## Conclusion

The spatial distribution of CL incidence had cluster pattern in three northeastern provinces of Iran. In the southern regions of North Khorasan Province and northern areas of Razavi Khorasan Province, it makes hot spots and the CL incidence is a function of spatial and geographical trends. In addition, spatial trends in the disease incidence distribution indicate that it is not greatly increased or decreased from one area to another. It appears as hot spots areas. Spatial analysis by showing high-risk areas can be useful tools for controlling and preventing CL incidence.

## Ethical considerations

Ethical issues (Including plagiarism, informed consent, misconduct, data fabrication and/or falsification, double publication and/or submission, redundancy, etc.) have been completely observed by the authors.
